# Contingency inferences from base rates: A parsimonious strategy?

**DOI:** 10.3758/s13421-024-01567-y

**Published:** 2024-05-06

**Authors:** Niklas Pivecka, Moritz Ingendahl, Linda McCaughey, Tobias Vogel

**Affiliations:** 1 Department of Occupational, Economic, and Social Psychology, University of Vienna, Universitätsstr. 7, A-1010 Vienna, Austria; 2 Department of Psychology, Ruhr University Bochum, Bochum, Germany; 3 Faculty of Psychology, Dresden University of Technology, Dresden, Germany; 4 Department of Economic Psychology, Darmstadt University of Applied Science, Darmstadt, Germany

**Keywords:** Base rates, Contingency learning, Ecological correlation, Probability judgment, Pseudocontingency, Top-down

## Abstract

The pseudocontingency framework provides a parsimonious strategy for inferring the contingency between two variables by assessing the base rates. Frequently occurring levels are associated, as are rarely occurring levels. However, this strategy can lead to different contingency inferences in different contexts, depending on how the base rates vary across contexts. Here, we examine how base-rate consistency influences base-rate learning and reliance by contrasting consistent with inconsistent base rates. We hypothesized that base-rate learning is facilitated, and that people rely more on base rates if base rates are consistent. In Experiment 1, the base rates across four contexts implied the same (consistent) or different (inconsistent) contingencies. Base rates were learned equally accurately, and participants inferred contingencies that followed the base rates but deviated from the genuine contingencies within contexts, regardless of consistency. In Experiment 2, we additionally manipulated whether the context was a plausible moderator of the contingency. While we replicated the first experiment's results when the context was a plausible moderator, base-rate inferences were stronger for consistent base rates when the context was an implausible moderator. Possibly, when a moderation-by-context was implausible, participants also relied on the base-rate correlation across contexts, which implied the same contingency when base rates were consistent but was zero when the base rates were inconsistent. Thus, our findings suggest that contingency inferences from base rates involve top-down processes in which people decide how to use base-rate information.

**Supplementary Information:**

The online version contains supplementary material available at 10.3758/s13421-024-01567-y.

## Introduction

Detecting the contingency between events is essential for making informed decisions and predictions (Crocker, 1981). Genuine contingency estimation involves assessing the joint frequencies of two binary events (Allan, 1980). For instance, consider a voter evaluating their political alignment with a party. The voter might compare specific policies they support with the specific policies endorsed by the party, quantified as Δ*p* = *p*(Policies supported by voter | Policies supported by Party A) – *p*(Policies supported by voter | Policies opposed by Party A) (Jenkins & Ward, 1965). In this example, the voter assesses each policy, gauging their support or opposition, and compares it with the party’s stance. However, while this strategy may be useful for a few key policies, it becomes cumbersome with multiple policies across diverse domains. Further, voters may often rely on aggregated data, such as the overall sentiment and topics of discussion related to a party (e.g., Party A advocates for reducing most taxes). Thus, constraints such as limited memory capacity and ecological factors may hinder individuals from approximating the genuine contingency algorithm, and a different strategy is necessary for inferring contingencies in such situations (Fiedler et al., 2009).

When individuals cannot infer genuine contingencies due to memory constraints or other factors, they may rely on the skewed base rates of the levels of a pair of variables (Fiedler & Freytag, 2004). This approach suggests that people associate frequently occurring levels with each other, and by implication, infrequently occurring levels as well (Kutzner et al., 2011). For example, if both the voter and the party predominantly support most environmental policies, the voter should infer that there is a high degree of agreement between their stances. Conversely, if the voter generally supports most environmental policies while the party opposes them, the voter should infer a discrepancy in their policy support.^1^

**Fig. 1:**
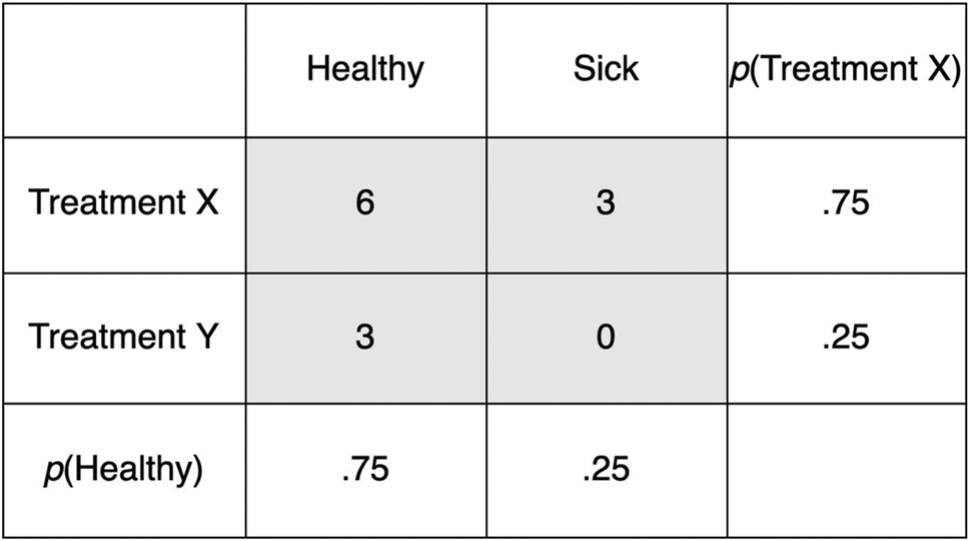
Pseudocontingencies in single contexts. The genuine contingency shows that Treatment X is less likely to improve health than Treatment Y, Δ*p* = (healthy | Treatment X) – (healthy | Treatment Y) = 6/9 – 3/3 = -.33. However, the frequent occurrence of both Treatment X and improved health outcomes can create the misleading impression that Treatment X is more likely to improve health

Intriguingly, such inferences from base rates do not require individuals to encode the joint frequencies of two variables. Instead, they only need to encode the base rates of each variable. In other words, the cognitive process underlying base rate inferences does not reflect the genuine contingency algorithm, and these so-called *pseudocontingencies* can sometimes lead to flawed conclusions (Fiedler & Freytag, 2004). For example, Fig. 1 illustrates the contingency between a medical treatment (patients receive either Treatment X or Treatment Y) and a subsequent health condition (patients are either healthy or sick). Although Treatment X and improved health are both frequent, calculating the genuine contingency shows that Treatment Y (i.e., the less frequent treatment) is, in fact, more likely to improve health. Such instances highlight the potential pitfalls of relying solely on base rate inferences for contingency detection.

Many experiments have shown that people rely on base rates to infer contingencies, even when the genuine contingency is zero or when its sign is the opposite of what the base rates suggests (e.g., Eder et al., 2011; Ernst et al., 2019; Fiedler, 2010; Vogel & Kutzner, 2017). However, pseudocontingencies should not be dismissed as mere misperceptions. Instead, they can be seen as examples of adaptive cognition (Fiedler et al., 2013). Although base rates do not determine joint frequencies, they impose constraints and limit the possible range of contingencies (cf., Fiedler et al., 2013; Vogel & Kutzner, 2017). Complementing this argument, simulations show that when two levels frequently occur in a sample, it typically indicates that both levels are associated on a population level (Kutzner et al., 2011). Since tracking and remembering base rates is easier than assessing joint frequencies, pseudocontingencies represent a parsimonious strategy for detecting contingencies in real-world ecologies with abundant information (Fiedler et al., 2009, 2013).

So far, we have focused on how people infer contingencies from base rates in single contexts. However, the pseudocontingency framework also applies to situations where individuals assess the contingency between two variables across multiple contexts (e.g., Bott & Meiser, 2020; Fiedler et al., 2007). For example, Treatments X and Y may be used for multiple diseases, raising the question of how people use base-rate information in such situations.

### Pseudocontingencies in multiple contexts

The same process that applies in single contexts can also be extended to multiple contexts. People can detect the base rates in each context and infer contingencies conditional on the context. Recent research by Vogel et al. (2022) demonstrated that people infer up to four conditional contingencies from contexts with skewed base rates. In one of their experiments, participants were asked to judge the contingency between treatment (X and Y) and outcome (health improved and worsened) of patients suffering from one of four diseases (A, B, C, and D), which represented the contexts. For each disease, participants viewed the treatment and outcome for 12 patients. For two diseases, base rates of Treatment X and improved health were skewed in the same direction – when most patients received Treatment X, health improved for most patients, and vice versa. In the remaining two diseases, the base rates of Treatment X and improved health were skewed in the opposite direction – when most patients received Treatment X, health worsened for most patients, or when most patients did not receive Therapy X, health improved for most patients. Hence, the base rates of Treatment X and improved health implied different contingencies depending on the context. Participants judged that Treatment X (vs. Y) was more likely to improve health when Treatment X and improved health were (in)frequently occurring. Conversely, participants judged that Treatment Y (vs. X) was more likely to improve health when Treatment Y and improved health were (in)frequently occurring. Contrary to these judgments, the actual sign of the contingency in each context was in the opposite direction of what the base rates implied (Vogel et al., 2022; Experiment 1). Thus, it appears that people assess base rates in each context individually and infer conditional contingencies. However, assessing pairwise base rates in every context, storing them in memory, and inferring the contingency conditional on the context can become a very demanding memory task itself as the number of contexts increases (see also Vogel, Freytag, et al., 2013).

The concept of pseudocontingency is rooted in the idea of parsimony, which suggests that people prefer simple representations (Fiedler et al., 2009, 2013). Thus, it may be premature to assume that individuals will always infer contingencies by assessing base rates individually for each context, as this would severely limit the potential of pseudocontingencies as a parsimonious yet fairly accurate alternative to genuine contingencies (Fiedler et al., 2013). Instead, people may attempt to store base-rate information more efficiently by organizing more information in single memory chunks, reducing cognitive demands, and potentially facilitating base-rate learning (for literature on chunking and working memory capacity, see Mandler, 2011; Miller, 1956; Thalmann et al., 2019). However, previous research has not provided a clear answer about the circumstances under which this would be the case. In the following sections, we propose that base-rate learning is either facilitated or impeded depending on how the base rates vary across contexts. We also propose that how the base rates vary across contexts influences how much people rely on base rates when making contingency judgments. Finally, we propose that people adopt a top-down decision-making process, determining whether the situation warrants a simple or complex representation of base-rate information.

### Base-rate learning in multiple contexts

**Fig. 2:**
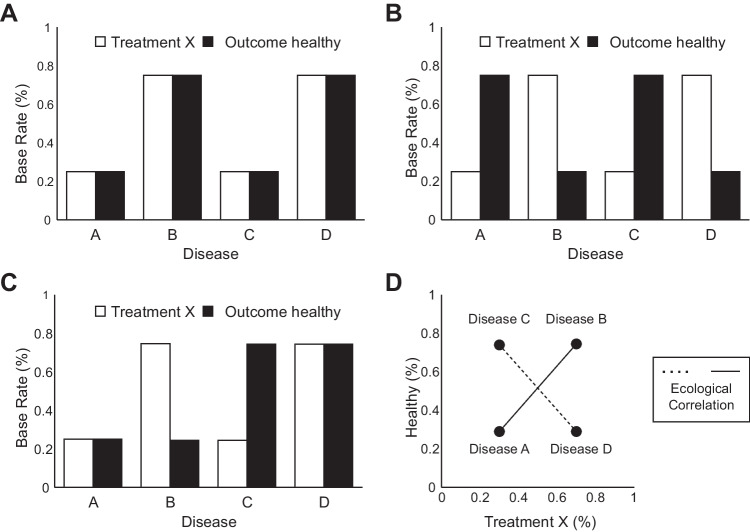
Consistent versus inconsistent base rates across contexts. (**A**) Scenarios where the base rates for Treatment X and improved health are consistently either high or low across all four diseases, suggesting that both levels are associated in each context. (**B**) Scenarios with diverging base rates for all four diseases, where either Treatment X is frequent and improved health is infrequent, or vice versa. (**C**) A mix of these patterns: for two diseases, base rates for Treatment X and improved health are the same (either both frequent or infrequent), while for the other two diseases the base rates are diverging, suggesting different treatment-outcome contingencies in each context. (**D**) Ecological correlations across contexts, with a positive ecological correlation between Treatment X and improved health for the distribution in Fig. 2A (solid line), a negative ecological correlation for both levels for the distribution in Fig. 2B (dashed line), and a zero ecological correlation for the distribution in Fig. 2C (both lines)

Previous studies on pseudocontingencies in multiple contexts typically exposed participants to scenarios where the base rates remained consistent across all contexts, that is, the skewed base rates always implied the same contingency in all targeted contexts (e.g., Fiedler & Freytag, 2004; Fleig et al., 2017; Meiser & Hewstone, 2004). Consider Fig. 2A and B, which depict the base rates for Treatment X and improved health in four diseases.

In Fig. 2A, low base rates for Treatment X correspond to low base rates of improved health, while high base rates of Treatment X correspond to high base rates of health improvement. Thus, base rates of Treatment X and improved health consistently imply that Treatment X and improved health should be linked. Conversely, Fig. 2B demonstrates scenarios where the frequencies of Treatment X and improved health outcomes diverge in all contexts. Thus, the base rates consistently imply that Treatment Y (and not X) and improved health should be linked. Notably, this is in contrast to the experiment by Vogel et al. (2022) introduced earlier, where participants encountered a mix of contexts where base rates of Treatment X and improved health were skewed in the same direction in some contexts but skewed in the opposite direction in the other contexts (see Fig. 2C).

At first glance, the difference may seem trivial as both consistent and inconsistent base rates across contexts produce the typical pseudocontingency effect: Frequent levels of a pair of variables are associated with each other even though this contradicts the genuine contingencies within contexts (Fiedler et al., 2007; Vogel et al., 2022). However, the representation of the base-rate information in memory, and the reliance and use of base-rate information for contingency judgments, may vary depending on the consistency of the base rates across contexts.

From an efficiency-based perspective, people should not store more information in memory than necessary for the task at hand. This is in line with research showing that people prefer to simplify information in memory by chunking it (Cowan et al., 2004) and that more information can be stored when the organization of information allows compression of data (Chekaf et al., 2016). When the base rates are consistent across contexts, redundant information is present. For instance, in Fig. 2A, people can summarize the base-rate information across contexts by learning that high Treatment X base rates coincide with high base rates of positive health outcomes, and low Treatment X base rates coincide with low base rates of positive health outcomes. A similar strategy is possible for Fig. 2B, where high Treatment X base rates coincide with low base rates of improved health (and vice versa) in all contexts. However, this summarization is not possible in Fig. 2C, where base rates of Treatment X and improved health are not consistently skewed in the same or opposite direction across contexts. Consequently, memory demands should be lower when facing contexts with consistent base rates, as this simplifies the process of learning and remembering base-rate information.

In most pseudocontingency experiments, not only are base rates consistent across contexts, they also vary systematically, creating contexts with opposing base rate trends, so-called *contrastive contexts* (Fiedler & Freytag, 2004). Figure 2A demonstrates contrastive contexts, where frequencies for both Treatment X and improved health increase from Disease A to B. Conversely, in Fig. 2B, while the frequency of Treatment X increases, the frequency of improved health decreases from Disease A to Disease B. Fiedler and Freytag (2004) suggested that such contrastive contexts highlight the base rates across all targeted contexts and may therefore enhance base-rate learning even further. In comparison, in Fig. 2C, Diseases A and B do not constitute contrastive contexts, implying that the observed information in the second context cannot be stored as simple inversion of the first context but rather must be stored separately in memory. Therefore, consistent base rates across contexts and contrastive contexts should facilitate base-rate learning by allowing people to store information more efficiently. However, it has yet to be tested whether base rates are indeed learned more efficiently under such circumstances.

### Reliance on base rates for contingency judgments

Consistent base rates across contexts may not only facilitate the learning of base rates but also foster the reliance on base rates as proxy for genuine contingencies. Fiedler and Freytag (2004) originally assumed that pseudocontingencies in contexts with consistent base rates are facilitated because base rates are correlated at an aggregated level. This type of correlation, known as *ecological correlation*, refers to the relationship between the base rates of two variables across different contexts and can vary significantly from the individual correlation within each context (Robinson, 1950). For example, Fig. 2D illustrates a positive ecological correlation between the base rates of Treatment X and improved health between Diseases A and B, as they increase in tandem. However, it is crucial to note that this ecological correlation does not imply the same genuine contingency between Treatment X and improved health within each disease context. In fact, within these diseases, the contingency between Treatment X and improved health could be opposite to the ecological correlation, as illustrated before in Fig. 1.

The subsequent literature considered pseudocontingencies as a cognitive analogy to the ecological correlation, arguing that even in the case of single contexts, an ecological correlation may be inferred because the skewed base rates are compared to an implicit reference context (Fiedler et al., 2007, 2009). Thus, instead of inferring multiple conditional contingencies by evaluating and storing base rates for each context separately, people may infer a single unconditional contingency that follows the sign of the ecological correlation. The ecological correlation as the driver of pseudocontingencies also resonates with the idea that inferences from base rates represent a parsimonious strategy for contingency detection (Fiedler et al., 2009, 2013), as assessing the ecological correlation allows people to store all information in a single memory chunk that is updated with each newly observed context. However, in earlier experiments investigating pseudocontingencies in multiple contexts, it was not possible to distinguish between the base rates within contexts and the ecological correlation as possible sources of information for contingency inferences because both sources always implied the same contingency (e.g., Fiedler et al., 2007; Fiedler & Freytag, 2004; Meiser & Hewstone, 2004).

The idea that the ecological correlation is the main or even exclusive driver of pseudocontingencies was challenged by the findings of Vogel et al. (2022). In their experiments, the ecological correlation was held constant at zero. Yet, participants still estimated that frequently occurring levels are associated in each context. Thus, participants inferred four conditional contingencies, indicating that base rates within contexts, independent of the ecological correlation, are sufficient to create pseudocontingency effects. Nevertheless, when both within-context base rates and ecological correlation suggest the same contingency, they might have a combined effect on contingency judgments.

Recognizing that the contingency is the same in each context based on consistent base rates may reinforce people’s confidence in their own judgments. Similar to confirmatory hypothesis testing (Klayman & Ha, 1987; Nickerson, 1998), people may form a contingency judgment based on their initial observations and attempt to confirm it in subsequent contexts. If their judgment is based on the base rates, it will be consistently corroborated when the base rates remain consistent across all contexts, but it will be challenged when the base rates are inconsistent. Further, experiencing consistent base rates in every context and recognizing that contrastive contexts generate an ecological correlation that implies the same contingency sign as the base rates within contexts, may further confirm people’s judgments that the frequent level of a pair of variables are associated. Put differently, regardless of whether contingencies are inferred conditional on the context or unconditionally, consistent base rates across contexts should amplify the effect of pseudocontingencies.

### Conditional or unconditional? Causal assumption about the context

The observation that people tend to rely mainly on base rates within contexts to infer conditional contingencies also raises a novel question that has not yet been addressed: *When* do individuals detect and consider the context as a plausible moderator of the contingency? Relying on the within-context base rates suggests that individuals assume the contingency varies across contexts, making it inappropriate to rely on the ecological correlation as a more parsimonious but less accurate representation of the base-rate information. This entails a top-down process in which people must evaluate the observed situation and decide whether the context is a plausible moderator.

To some extent, base-rate learning is a bottom-up process in which people observe and store base-rate information from their environment. However, base-rate learning is also influenced by top-down processes in which people direct their attention to information according to prior beliefs (for the influence of prior beliefs on learning and perception, see Baluch & Itti, 2011; Kok et al., 2013). Ecological approaches to learning suggest that people learn out of necessity (e.g., Gibson, 1966, 1976). Thus, as people learn, they must anticipate how the base-rate information may be useful in a later decision or judgment. There are two aspects to consider in this top-down process.

First, people may directly infer from the base rates whether a moderation-by-context is plausible. If the base rates vary inconsistently across contexts, signaling different contingency signs, people may recognize that the context may serve as a plausible moderator. In contrast, when the base rates are consistent across contexts, people may regard the context as an implausible moderator and instead resort to a more parsimonious representation by relying on the ecological correlation for contingency judgments.

Second, people may have prior beliefs about whether the content of the context is a plausible moderator of the contingency. For example, it seems reasonable to assume that the effectiveness of a given treatment on patients' health depends on the specific disease. In such situations, individuals may be more inclined to consider the context as a plausible moderator. On the other hand, in scenarios where the context is more arbitrary, such as the effectiveness of a vaccine in different hospitals, individuals may be less likely to view the context as a plausible moderator. The question as to whether people judge contingencies conditional on the context or not has also been posed in other areas.^2^ Supporting the assumption that people strive for parsimony, people often disregard the context and rely on unconditional contingencies (e.g., Schaller & O’Brien, 1992). However, they also seem to consider the context and base their judgment on conditional contingencies if the relevance of the context is evident from the content (Spellman, 1996). Thus, depending on the content, the context may serve different functions in the contingency between two variables. In some causal models, the context may plausibly serve as a moderator of the contingency. In others, it might be implausible and, thus, should not be considered. Hence, judgments of contingencies may differ depending on the specific features of the setting and the causal model it implies.

## The present research

Previous research on contingency inferences from base rates has investigated scenarios where the base rates were either consistent across contexts (e.g., Fiedler & Freytag, 2004; Fleig et al., 2017; Meiser & Hewstone, 2004) or, more recently, inconsistent (Vogel et al., 2022). Here, we contrast both scenarios and investigate whether scenarios with consistent base rates across contexts lead to more pronounced pseudocontingency effects.

The first objective of this research was to investigate whether base-rate learning is facilitated when base rates remain consistent across contexts. We assumed that people strive for parsimonious representations and find encoding and storing base-rate information easier when the cognitive demands are reduced. Consistent base rates allow people to learn base rates more efficiently by summarizing redundant information and storing more information in single memory chunks. Additionally, contrastive contexts, which are particularly salient when the base rates are consistent, enable people to learn the base rates of one context as the mere inversion of the other context, again significantly reducing cognitive demands.

- H1: Base rates are recalled with higher accuracy when the base rates are consistent compared to inconsistent across contexts.

The second objective was to investigate whether people rely on base-rate information for contingency judgment to a greater extent when the base rates are consistent across contexts. Vogel et al. (2022) demonstrated that people infer conditional contingencies by assessing the base rate within contexts, even in the absence of an ecological correlation. However, we propose that this pseudocontingency effect will be even stronger when the base rates vary consistently across contexts. Thus, we hypothesized that participants express pseudocontingencies that deviate from the genuine contingencies within contexts, replicating Vogel et al. (2022), but that this pseudocontingency effect will be more pronounced when the base rates are consistent across contexts compared to inconsistent.

- H2: Pseudocontingencies that deviate from the genuine contingency within contexts will be more pronounced when the base rates across contexts are consistent than when they are inconsistent.

And as a final and third objective, we examined whether people rely more on the ecological correlation in situations where a moderation-by-context is implausible. We argue that this may already be the case when the base rates are consistent across contexts because consistent base rates signals that the contingency does not vary across contexts. However, a moderation-by-context should especially be ruled out when the content of the context makes a moderation implausible, and people should be more inclined to just follow the sign of the ecological correlation when they judge the same contingency across multiple contexts. In other words, people’s within-context contingency judgments should be more heavily influenced by the consistency of the base rates when a moderation is implausible.

- H3: The influence of consistent versus inconsistent base rates on the formation of pseudocontingencies is more pronounced when the content suggests that a moderation-by-context is implausible rather than plausible.

To test these hypotheses, we conducted two experiments in which participants were exposed to information about two binary variables across *four* contexts. For half of the participants, the base rates varied inconsistently across four contexts (see Fig. 2C). For the other half of the participants, the base rate varied consistently across the four contexts (see Fig. 2A and B). This design also ensured that the ecological correlation always implied the same contingency as the base rates within contexts for participants with consistent base rates but was always zero for participants with inconsistent base rates (see Fig. 2D). Importantly, the actual contingency within contexts was always contradicting the contingency implied by the base rates within contexts (see Table 1 for stimulus distribution). In Experiment 2, we additionally manipulated the content of the context, making a moderation-by-context plausible or implausible.

**Table 1:** Stimulus distributions across contexts in Experiments 1 and 2

	Context *z*				Pooled
	*z*_*1*_	*z*_2_	*z*_3_	*z*_4_	*z*_*1*_+* z*_2_+* z*_3_+* z*_4_
Cell frequencies					
*x*_1_/*y*_1_	6	3	3	0	12
*x*_1_/*y*_2_	3	6	0	3	12
*x*_2_/*y*_1_	3	0	6	3	12
*x*_2_/*y*_2_	0	3	3	6	12
Variable *x* base rate					
*p*(*x*_1_)	.75	.75	.25	.25	.5
*p*(*x*_2_) = 1 - *p*(*x*_1_)	.25	.25	.75	.75	.5
Variable *y* base rate					
*p*(*y*_1_)	.75	.25	.75	.25	.5
*p*(*y*_2_) = 1 - *p*(*y*_1_)	.25	.75	.25	.75	.5
Conditional probabilities					
*p*(*y*_1_|*x*_1_)	.67	.33	1.0	.0	.5
*p*(*y*_2_|*x*_1_)	1.0	.0	.67	.33	.5
Stimulus contingency					
Δ*p*	-.33	+.33	+.33	-.33	.0

In Context *z*_*1*_, *x*_1_ is jointly observed six times with *y*_1_ and three times with *y*_2_. *x*_2_ is jointly observed three times with *y*_1_ and zero times with *y*_*2*_. Thus, *x*_1_ and *y*_1_ are frequent in Context *z*_*1*_, which suggests a positive contingency between *x*_1_ and *y*_1_. However, the actual contingency between *x*_1_ and *y*_1_ (Δ*p*) is negative in Context *z*_1_. In Context *z*_4_, *x*_1_ and *y*_1_ are infrequent, again suggesting a positive contingency, despite the actual contingency within contexts being negative. In Context *z*_2_ and *z*_3_, the frequencies of *x*_1_ and *y*_1_ diverge while the actual contingencies between *x*_1_ and *y*_1_ are positive. For Experiments 1 and 2, participants with inconsistent base rates across contexts experienced all four contexts, whereas participants with consistent base rates across contexts experienced either Context *z*_1_ and *z*_4_ (perfect positive ecological correlation) or Context *z*_2_ and *z*_3_ (perfect negative ecological correlation) twice

## Experiment 1

The first experiment sought to test how consistent (vs. inconsistent) base rates across contexts influences base-rate learning and reliance on base rates for contingency judgments. Participants’ task was to compare the responses of two politicians (A or B) in four contexts, which were different political domains. Both politicians answered statements in each domain with either “yes” or “no.” The contingency describes that a “yes” answer from Politician B is contingent on Politician A answering “yes” to the same statement. We adopted this scenario from previous research where it was successfully used to induce pseudocontingencies (Vogel et al., 2022, Experiment 1; Vogel, Freytag, et al., 2013, Experiment 3). We predicted that participants learn the base rates more accurately when base rates are consistent across the four political domains than when the base rates are inconsistent. We also predicted the typical pseudocontingency effect: participants should perceive pseudocontingencies (i.e., associating frequently occurring levels) that differ from the genuine contingencies within contexts. However, this pseudocontingency effect should be stronger when the base rates are consistent rather than inconsistent across contexts. Note that in the case of consistent base rates, the ecological correlation was always perfect, suggesting the same contingency as the base rates within contexts. In case of inconsistent base rates, the ecological correlation was always zero, implying no contingency and contradicting the base rates within contexts.

### Method

#### Design and participants

The study was conducted online in German language using Sosci-Survey (Leiner, 2014). A convenience sample of 81 participants (*M*_age_ = 21.2, *SD* = 2.6 years; 74 female) was acquired via social media with the prospect of winning a voucher for an online shop or receiving course credits. The design was a one-factorial between-subjects design with random assignment of participants to either consistent or inconsistent base rates across contexts. The sample size suffices for detecting an effect of Cohen’s *f* = 0.31 with 80% power in a one-way ANOVA (Faul et al., 2007). Participants who were exposed to contexts with inconsistent base rates experienced two contexts where the base rates of “yes” responses by Politicians A and B were skewed in the same direction and two contexts where they were skewed in opposite directions. Participants who viewed contexts with consistent base rates experienced four contexts where the base rates of Politician A’s and Politician B’s “yes” responses were either all skewed in the same direction, or all skewed in opposite directions (randomly counterbalanced between participants). The actual contingency within contexts was always opposite to what the base rates within-contexts implied, and the unconditional contingency pooled across contexts was always zero for all participants.

#### Materials and procedure

We used the same cover story as in Experiment 2 by Vogel et al. (2022). Two politicians, A and B, indicated their agreement (“yes”/“no”) with policy statements in four different domains (internal security, education, environment, and migration), which represented the contexts. Within domains, a “yes” response always indicated support for a specific type of policy. For example, if a politician answered “yes” to statements for environmental policies (e.g., “The deforestation of the rainforest must be stopped”), it was understood that the politician favored environmentally friendly policies. Conversely, a “no” response signified a lack of support for such policies. One domain was randomly selected, and 12 policy statements were presented alongside the answers given by Politician A. After the first domain, the remaining domains were displayed for Politician A in random order. Subsequently, participants were informed that another politician, Politician B, responded to the same survey, and participants studied the responses of Politician B in the same manner.

We manipulated the base rates of Politician A responding “yes” (in the following referred to as A base rate) and Politician B responding “yes” (in the following referred to as B base rate). Participants with inconsistent base rates across contexts experienced two policy domains where Politician A responded “yes” to most statements, *p* = .75, but two domains where Politician A responded “yes” to only a few statements, *p* = .25. Orthogonally, Politician B had low base rates of “yes” responses in two policy domains, *p* = .25, but high in the other two domains, *p* = .75. Participants with consistent alignment across contexts also experienced two domains with high and low A and B base rates. However, the base rates were not orthogonal to each other. That is, Politician A’s and Politician B’s “yes” responses were always skewed in the same or opposite direction.

After participants had studied the responses of both politicians for all four domains, they indicated the A and B base rates in each domain. Specifically, for each domain, they provided a base rate estimate for each politician by indicating what percentage of statements in that domain the politician agreed with. Then, participants provided estimates of the contingency between the politicians’ answers for each domain. For example, they read, “For a statement on education which Politician A answers with *yes*, Politician B would probably answer with…” and provided their estimate by moving a slider on 101-point scale with anchors ranging from “definitely no” (coded 0) to “definitely yes” (coded 1). Participants also provided the estimate conditional on that Politician A had answered the statement with “*no*” (“For a statement on education that Politician A answered with *no*, Politician B would probably answer with…”), using the same scale. We used the estimates to calculate domain-wise contingency estimates, Δ*p*, with a theoretical range from -1 to +1 by subtracting the latter estimates from the former. Finally, participants provided demographic information before they were debriefed and thanked.

### Results and discussion

#### Base rate learning

Our first hypothesis was that base-rate learning is facilitated when base rates vary consistently (vs. inconsistently) across contexts. We expected that participants who experienced consistent base rates would estimate higher base rates when the actual base rates of Politician A's and Politician B's “yes” statements were high, and lower base rates when the actual base rates were low, than participants who experienced inconsistent base rates.^3^

We aggregated base-rate estimates across contexts and politicians and then subjected the base-rate estimates to a 2(actual base rate: high vs. low) × 2(consistency: consistent vs. inconsistent) mixed ANOVA. In our main analysis, along with *p-*values, we also report Bayes factors. These were calculated using the *BayesFactor* package, adhering to the default priors (Morey & Rouder, 2018). We always report the strength of evidence in favor of the alternative hypothesis compared to the null hypothesis.

The ANOVA revealed a significant main effect of the actual base rates on base-rate estimates, *F*(1, 79) = 55.05, *p* < .001, Cohen’s *f* = 0.84, *BF*_10_ > 1000. Participants estimated higher base rates when the actual base rates were high (*M* = 56.69, *SE* = 1.58) than when the actual base rates were low (*M* = 43.69, *SE* = 1.40). However, the interaction between actual base rates and consistency was not significant, *F*(1, 79) = 0.51, *p* = .477, Cohen’s *f* = 0.08, *BF*_10_ = 0.32. Thus, we obtained no evidence that base rates were learned more accurately when the base rates were consistent across context. The main effect of consistency was also not significant, *F*(1, 79) = 0.90, *p* = .346, Cohen’s *f* = 0.11, *BF*_10_ = 0.28.

#### Contingency judgments

Our second hypothesis was that participants express pseudocontingencies (i.e., contingencies that are the opposite sign of the genuine contingency within contexts) more strongly when the base rates are consistent across contexts. To test this hypothesis, we aggregated the domain-wise contingency estimates across contexts to obtain one score per participant representing the belief in a pseudocontingency. For participants with inconsistent base rates across contexts, the contingency estimates for the two contexts with oppositely skewed base rates of Politician A's and Politician B's “yes” responses were subtracted from the contingency estimates for the contexts with base rates of Politician A's and Politician B's “yes” responses skewed in the same direction and divided by the total number of contexts. For participants with consistent base rates across contexts, we averaged the contingency estimates for all four contexts and reversed the score for participants who always experienced contexts with base rates skewed in the opposite direction (i.e., multiplied by -1). Thus, for all participants, positive values indicate a belief in the direction of the pseudocontingency, whereas negative values indicate a belief in the direction of the genuine contingency. More specifically, if participants correctly identified the genuine contingency in each context, the pseudocontingency score would be -.33.

**Fig. 3:**
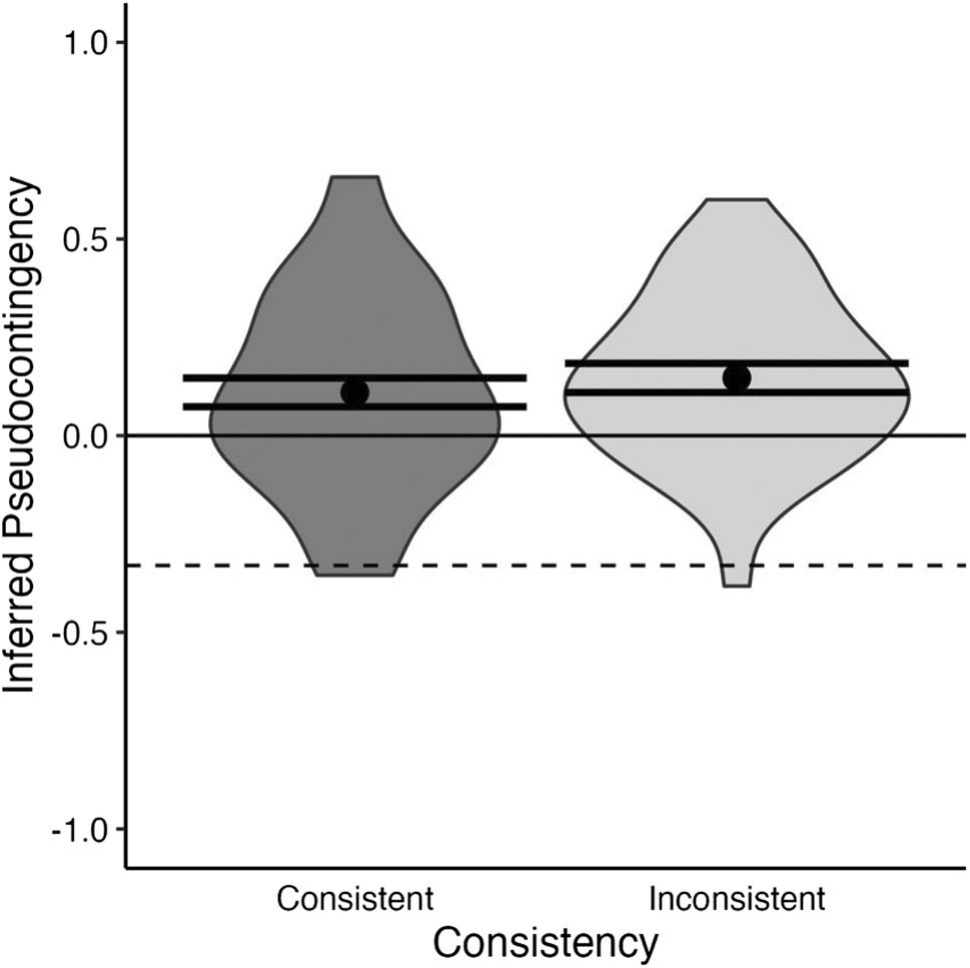
Average inferred pseudocontingency for consistent and inconsistent base rates in Experiment 1. The dashed horizontal line represents the objective value of the genuine contingencies at -.33, whereas the solid horizontal line represents a zero contingency between Politician A’s and Politician B’s “yes” responses. Error bars indicate standard errors and grey areas display kernel densities.

We conducted a one-factor ANOVA to examine the differences in mean pseudocontingency scores when base rates were consistent or inconsistent across contexts. The results revealed that there was no statistically significant difference in mean pseudocontingency scores when base rates were consistent (*M* = 0.11, *SE* = 0.04) versus inconsistent (*M* = 0.15, *SE* = 0.04), *F*(1,79) = 0.50, *p* = .482, Cohen’s *f* = 0.08, *BF*_10_ = 0.29. The mean contingency estimates in both conditions exceeded the genuine contingencies of -.33 (*t*s > 12.03, *p*s < .001, *BF*_10_s > 1000), and were also significantly higher than zero (*t*s > 3.00, *p*s < .004, *BF*_10_s > 4.88). Hence, participants in both conditions exhibited a pseudocontingency (see Fig. 3).

To gain a better understanding of the results, we also examined the domain-wise contingency estimates by conducting a multi-mixed model analysis (Snijders & Boskers, 2012). This involved subjecting the domain-wise contingency estimates to a three-way ANOVA with the factors A base rates, B base rate, and consistency. Detailed model results along with estimated marginal means and visualization are reported in the Online Supplementary Materials (OSM). The analysis revealed that participants tended to perceive a positive contingency between Politician A’s and Politician B’s “yes” responses when base rates of “yes” responses were either high or low for both politicians. Conversely, estimated contingencies tended to be negative when one politician had high base rates of “yes” responses but the other had low base rates of “yes” responses. This main effect of within-context base rates on contingency judgments was independent of the consistency of base rates across contexts.

#### Discussion

In Experiment 1, we did not find that base-rate learning was facilitated when the base rates were consistent across contexts, contrary to our expectations. Further, the consistency of the base rates across contexts in Experiment 1 did not exert a significant influence on the contingency estimates. That is, base rates within contexts had the same effect on contingency estimates when the base rates were consistent or inconsistent across contexts. Thus, our results replicate the findings of Vogel et al. (2022) and imply that base rates within contexts alone are sufficient to drive contingency inferences and that people tend to infer different contingencies as a function of the context.

Arguably, the content in Experiment 1 made a moderation-by-context very plausible. This may have promoted an inference strategy that allows for conditional contingency estimates. It does not seem far-fetched to assume that politicians’ agreement on policies may depend on the specific policy domain. Thus, participants may have had the prior belief that the contingency varies across contexts and assumed that disregarding the context and relying on the ecological correlation would be associated with a loss of accuracy.

Research indicates that contingency detection reacts to assumptions about causal relations among variables. In this vein, assumptions about causal relations between two events affect how covariation information is weighted (Fugelsang & Thompson, 2000, 2003; Mata et al., 2015). Moreover, if one event plausibly causes the other event, participant actively seek positive evidence for a contingency between both events (Goedert et al., 2014). Accordingly, several scholars have proposed propositional models to model logical rules and causal relations (De Houwer, 2014; Mitchell et al., 2009; Novick & Cheng, 2004; Waldmann & Holyoak, 1992). Yet, little is known as to whether the context would affect inferential strategies. In Experiment 2, we therefore examined the role of context plausibility as a moderator of contingency inferences to investigate one of the potential determinants of when people prefer conditional to unconditional inferences.

## Experiment 2

In Experiment 2, we varied how plausible it is that a given context moderates the contingency. We predicted a replication of the previous findings – pseudocontingencies that deviate from the genuine contingencies within contexts – if a moderation is plausible. If a moderation is *not* plausible, participants may infer a single contingency judgment rather than conditional contingency inferences. Then, the consistency may influence the strength of the pseudocontingencies since the ecological correlation implies a zero contingency when the base rates were inconsistent but implies the same contingency when the base rates were consistent across contexts.

### Method

#### Design and participants

Two-hundred participants (*M*_age_ = 32.5, *SD* = 11.8 years; 120 female, 78 male, one diverse, one undisclosed) were acquired via a commercial panel provider and paid £1.50 for participation in a series of unrelated studies. This study adopted the medical treatment scenario from Vogel et al. (2022, Experiment 1). Participants’ task was to judge the contingency between Treatment X (vs. Y) and improved (vs. worsened) health condition. The design was the same as in the previous study, with one additional between-participant factor manipulating the plausibility of a moderation-by-context. This resulted in a 2(consistency: consistent vs. inconsistent) × 2(moderation plausibility: low vs. high) between-subjects design. The sample size suffices for detecting a small-to-medium interaction effect of Cohen’s *f* = 0.20 with 80% power in a standard 2 × 2 ANOVA (Faul et al., 2007).

#### Materials and procedure

Participants observed a series of patient data and were required to judge the contingencies between treatment (X vs. Y) and outcome (health improved vs. worsened) in four contexts. In the first phase, participants saw a list of patient IDs (e.g., “XHHOI3798V” or “VNVIG6689S”) and which treatment (“Medicine X” and “Medicine Y”) the patients received. This procedure was repeated for all four contexts. In the second phase, participants saw a list of the same patients, but now the information next to the patient ID indicated whether the health condition of the patient improved or worsened (e.g., “XHHOI3798V got better”; “VNVIG6689S got worse”). Again, this was repeated for the remaining three contexts. Then, participants continued with the base rate estimations. Participants estimated the base rate of Treatment X (vs. Y), and the base rate of patients with improved (vs. worsened) health condition. Afterwards, participants judged the treatment-outcome contingency by indicating the probability that the condition improved versus worsened given that a patient was treated with X, or treated with Y, respectively. This was measured on a 101-point sliding scale with the endpoints labelled “The patient’s condition will most likely get worse” (coded 0) and “The patient’s condition will most likely get better” (coded 1). The difference between these two estimates represents the context-wise contingency estimates, *Δp*. In the end, participants reported their demographics, were thanked, and debriefed.

Identical to Experiment 1, the base rates varied across contexts between participants (consistent versus inconsistent). In addition, the scenarios varied between participants to manipulate the plausibility that the context moderated the treatment-outcome contingency. For high moderation plausibility, participants were told to judge the treatment-outcome contingency for four different diseases (Morbus Alpha, Beta, Gamma, & Delta) serving as contexts. For the low moderation plausibility, participants were informed that patients from a certain hospital were suffering from one disease which was treated with Treatment X vs. Y. Different folders organized by patient surnames (Folder 1: A–G; Folder 2: F–K; Folder 3: L–O; Folder 4: P–Z) served as contexts. Hence, participants in the high plausibility condition judged the treatment-outcome contingency for the four diseases (e.g., “What will happen if Medicine X is given to a patient with Morbus Alpha?”), whereas participants in the low plausibility condition judged the treatment-outcome contingency for the four folders (“What will happen if Medicine X is given to a patient in Folder 1: A–G?)”.

The two scenarios had been selected due to findings from a pilot study in which participants (*N* = 45) read different medical treatment scenarios and hypothetical findings. For each hypothetical finding, they rated its plausibility on a scale ranging from 1 (not plausible at all) to 6 (highly plausible). The pilot revealed that a hypothetical main effect (“The type of treatment has an effect on the health condition.”) was considered highly plausible in both scenarios, *M*_high moderation plausibility_ = 5.60, *SD* = 0.67, *M*_low moderation plausibility_ = 5.51, *SD* = .92; *t*(44) = 0.66, *p* = .51, *BF*_10_ = 0.20. Regarding a moderation by context, participants found it very plausible that “For some of the diseases, Treatment X yields a better outcome, but for other diseases, Treatment Y yields a better outcome,” *M*_high moderation plausibility_ = 5.64, *SD* = 0.68. But it was considered much less plausible that “For some patients (e.g., surnames A-G), Treatment X yields a better outcome, for other patients (e.g., surnames L-O), Treatment Y yields a better outcome,” *M*_low moderation plausibility_ = 2.87, *SD* = 1.90, *t*(44) = 8.53, *p* < .001, *BF*_10_ > 1000). Except for the scenario and consequential rewording of conditional probability estimates, materials and procedure did not differ between conditions (sample materials can be viewed on the Open Science Framework at: https://osf.io/bxaqy/).

### Results and discussion

#### Base-rate learning

To investigate whether participants accurately learned base rates, we subjected aggregated base rate estimates to a 2(actual base rate: high vs. low) × 2(consistency: consistent vs. inconsistent) × 2(moderation plausibility: plausible vs. implausible) mixed ANOVA. The ANOVA revealed a highly significant main effect of the actual base rates on base rate estimates, *F*(1, 196) = 181.37, *p* < .001, Cohen’s *f* = 0.96, *BF*_10_ > 1000. Estimates were higher if stimulus base rates were high (*M* = 60.54, *SE* = 1.19), than if they were low (*M* = 36.78, *SE* = 1.04). However, we obtained no evidence that participants learned base rates more accurately depending on the consistency of the base rates across contexts, *F*(1, 196) = 0.34, *p* = .562, Cohen’s *f* = 0.04, *BF*_10_ = 0.19. No other effects, including the main effect of or any interaction with moderation plausibility, were significant in this model, *F*s < 1.04, *p*s > .31, *BF*_10_s < 0.21.

#### Contingency judgments

We again calculated the pseudocontingency score per participant from the domain-wise contingency estimates and subjected the scores to a 2(consistency) × 2(moderation plausibility) between-subject ANOVA. Here, the two-way interaction was significant, *F*(1, 196) = 4.73, *p* = .031, Cohen’s *f* = 0.16. However, the evidence in favor of the alternative hypothesis was only inconclusive to weak, *BF*_10_ = 1.69. None of the main effects were significant, all *F*’s < 2.55, all *p*’s > .112, *BF*_10_’s < 0.54.

When the context was a plausible moderator of the contingency, there was no significant difference in the pseudocontingency score when the base rates were consistent or inconsistent (*M*_consistent_ | _plausible_ = 0.10, *SE* = 0.05; *M*_inconsistent_ | _plausible_ = 0.13, *SE* = 0.05). In both cases, the pseudocontingency estimates exceeded the genuine contingencies within contexts of -.33 (*t*s > 8.04, *p*s < .001, *BF*_10_s > 1000). The pseudocontingency score was also significantly different from zero when the alignment was inconsistent across contexts, *t*(196) = 2.51,* p* = .013, *BF*_10_ = 7.04. For consistent alignment, the pseudocontingency score just failed to reach the conventional significance level, *t*(196) = 1.85, *p* = .066, *BF*_10_ = 0.94.

**Fig. 4:**
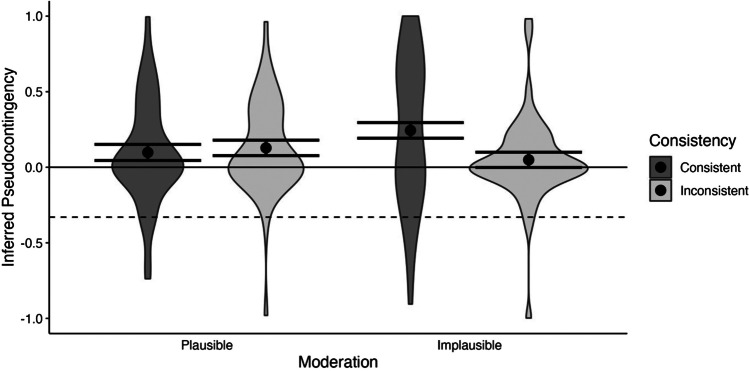
Average inferred pseudocontingency for consistent and inconsistent base rates and plausible and implausible moderation in Experiment 2. The dashed horizontal line represents the objective value of the genuine contingencies at -.33, whereas the solid horizontal line represents a zero contingency between Treatment X and improved health. Error bars indicate standard errors and grey areas display kernel densities

However, when the context was not a plausible moderator of the contingency, participants were more likely to perceive pseudocontingencies when the base rates were consistent (*M*_consistent_ | _implausible_ = 0.24, *SE* = 0.05) than when the base rates were inconsistent (*M*_inconsistent_ | _implausible_ = 0.05, SE = 0.05). While both estimates were significantly different from the value of -.33 for genuine contingencies (*t*s > 7.49, *p*s < .001, *BF*_10_s > 1000), only when the alignment was consistent across contexts did the pseudocontingency scores exceed chance level, *t*(196) = 4.73, *p* < .001, *BF*_10_ = 29.37. When the alignment was inconsistent, the estimate was not significantly different from zero, *t*(196) = 0.97, *p* = .331, *BF*_10_ = 0.32 (see Fig. 4).

A subsequent multi-mixed level model analysis supported that participants inferred positive contingencies between Treatment X and improved health when both levels were frequent or infrequent but inferred negative contingencies between Treatment X and improved health when one level was frequent while the other was infrequent. However, this effect depended on the consistency and the moderation plausibility. For implausible moderation, within base rates only predicted contingency estimates when base rates where consistent rather than inconsistent (for more details, see OSM).

Given the weak evidence for the interaction effect between consistency and plausibility in our main analysis, we descriptively analyzed how many participants in every group of our 2 × 2 design perceived a contingency in the direction of the pseudocontingency and how many a contingency in the direction of the genuine contingency. When moderation was plausible, 61.7% of participants with consistent base rates exhibited a pseudocontingency, while 38.3% recognized the genuine contingency. A similar trend was observed in participants with inconsistent base rates, with 62.8% identifying a pseudocontingency and 37.2% estimating a contingency in the direction of the genuine contingency. In conditions where moderation was deemed implausible, 62.0% of participants with consistent base rates identified a pseudocontingency, whereas 38.0% recognized the genuine contingency. Interestingly, when base rates were inconsistent, only 53.9% identified a pseudocontingency, 1.9% indicated no contingency, and 44.2% recognized the genuine contingency. Thus, the moderation plausibility had a noticeable impact on how participants judged contingencies in scenarios with consistent and inconsistent base rates.

Importantly, when the base rates were consistent, the ecological correlation always implied the same contingency as the alignment within base rates. In contrast, when the base rates were inconsistent, the ecological correlation was zero. Thus, the interaction effect between consistency and moderation plausibility may indicate that participants followed the sign of the ecological correlation to judge contingencies. To provide some indirect evidence for this, we calculated the subjective ecological correlation for each participant based on participants’ base rate estimates.^4^

In an exploratory analysis, we subjected contingency estimates (i.e., context-wise Δ*p*s) to a linear mixed model with the subjective ecological correlation as continuous predictor, and moderation plausibility as between-subject factor and permitted random intercepts for participants. The ecological correlation was grand mean centered, and we used effect coding for the moderation plausibility (-1 = plausible, 1 = implausible). The linear mixed model yielded a significant main effect of the ecological correlation, *b* = 0.27 (*SE* = 0.03), *t*(196) = 8.47, *p* < .001. Participants were more likely to estimate a positive contingency between Treatment X and improved health, when they also perceived a positive ecological correlation between both levels. Most importantly, there was also significant interaction effect between the ecological correlation and moderation plausibility, *b* = -0.09 (*SE* = 0.03), *t*(196) = -2.82, *p* = .005. The influence of the ecological correlation was more pronounced when moderation was implausible rather than plausible (for a visualization of the interaction effect, see Fig. S3 in the OSM). The main effect of the moderation plausibility was not significant, *b* = -0.03 (*SE* = 0.02), *t*(196) = -1.38, *p* = .168. Thus, the exploratory analysis supports our argument that participants may resort to unconditional contingencies by following the sign of the ecological correlation when a moderation-by-context is deemed implausible.

#### Discussion

The results from the last experiment replicate the effect of base rates within contexts on contingency judgments observed in the previous experiment and previous literature (Vogel et al., 2022). However, the base rates within contexts were no longer sufficient to drive contingency inferences if a moderation by context was not plausible. Under those circumstances, base rates only drove the judgment if they were consistent across contexts, so that the judgments within contexts were also implied by the ecological correlation across contexts.

## General discussion

The current research investigated how people infer contingencies from base rates in multiple contexts, and how the consistency of the base rate across contexts affects these base rate inferences. The pseudocontingency framework suggests that people associate frequently occurring levels of a pair of (binary) variables with each other, regardless of their joint frequencies (Fiedler & Freytag, 2004). In two experiments, we found that people perceived a pseudocontingency that was in the opposite direction of the genuine contingencies within contexts. Whether or not base rates were consistent across contexts affected base rate inferences only when the content of the context made a moderation-by-context implausible. In this case, contingency inferences were more likely to reflect the correlation of base rates across contexts (i.e., the ecological correlation), implying that participants relied on unconditional rather than conditional contingencies. Taken together, these findings enrich the pseudocontingency framework (Fiedler et al., 2009, 2013) by highlighting that the source of information people use to infer contingencies from base rates may depend on a top-down process. There, people use prior beliefs to decide whether or not the context is a plausible moderator of the contingency.

### Consistent versus inconsistent base rates across contexts

Throughout this paper, we referred to consistent base rates when the base rates of two variables implied the same contingency in each targeted context. Conversely, we referred to inconsistent base rates when the base rates implied different contingencies. Pseudocontingencies in multiple contexts have been investigated in scenarios where base rates were consistent across contexts (e.g., Fiedler & Freytag, 2004; Fleig et al., 2017; Meiser & Hewstone, 2004) and, more recently, when base rates were inconsistent across contexts (Vogel et al., 2022).

The primary goal of our research was to examine the effect of base rate consistency on base-rate learning and reliance for contingency judgments. In both experiments, we found no evidence that base rates are learned more accurately when base rates are consistent across contexts. Arguably, the memory task (i.e., learning pairwise base rates in four contexts) was not very difficult, allowing participants with inconsistent base rates across contexts to compensate for the higher memory demands by increasing their effort. The role of effort has recently been discussed in the limited-resource literature as a reason for inconsistent study results (Wright et al., 2019). According to the authors, fatigue from previous tasks may only affect performance on subsequent tasks if people conclude that they cannot successfully complete the task by increasing their effort (or do not consider it worthwhile to increase their effort). Thus, consistency may lead to more accurate base-rate learning when the task is more difficult (e.g., more than four contexts), so that people can no longer compensate by increasing their effort.

While participants did perceive pseudocontingencies in Experiment 1, we found no evidence that consistency leads to more pronounced pseudocontingency effect. Thus, we also obtained no evidence that within-context base rates and ecological correlation have an additive influence on contingency judgments when both sources imply the same contingency. Instead, our results replicate Vogel et al. (2022) by showing that within-context base rates are sufficient to create pseudocontingencies. However, we still advocate caution in completely dismissing the ecological correlation as an explanation for base rate inferences. Instead, our findings in Experiment 2 suggest that people may actively switch from conditional to unconditional contingency inferences when the situation warrants such a simplification – depending on prior beliefs.

### Pseudocontingencies: Bottom-up and top-down processes

Inferences from base rates are partially a bottom-up process as people observe and store base-rate information from their environment. Previous literature on pseudocontingencies was mostly concerned with this bottom-up aspect by investigated how skewed base rates affect contingency judgments and overwrite genuine contingencies (e.g., Fiedler, 2010). Only a few studies considered potential moderators of pseudocontingencies (Fleig et al., 2017), acknowledging the role top-down processes may play for inferences from base rates. Yet, the mere existence of two possible sources of information – base rates within contexts and ecological correlation – presents people with a decision as to which source to use. Here, the present research suggests that prior beliefs about whether the situation warrants a context moderation or not influence whether people infer conditional contingencies by using the base rates within contexts or resort to unconditional contingencies by following the sign of ecological correlation.

As mentioned in the interim discussion, the content in Experiment 1 made a moderation-by-context very plausible (i.e., politicians' agreement on policies may depend on the specific domain). The high plausibility of the context as a moderator of contingency may have led participants to judge each context separately, even though the base rates implied the same contingency in each context under consistent base rates. Thus, participants might have held a prior belief that the contingency varies across contexts. Consequently, they may have assumed that disregarding the context and relying on the ecological correlation would result in decreased accuracy. Therefore, in Experiment 2, we examined the role of context plausibility as a moderator of contingency inferences to investigate one of the potential determinants of when people prefer conditional to unconditional inferences. We found that when the context is a plausible moderator of contingency, context-specific base rates are a sufficient predictor of contingency judgments. However, when the context is not a plausible moderator, within-context base rates predicted contingency judgments only when the ecological correlation implied the same contingency.

Of particular interest is that participants used their prior beliefs to decide how to use base-rate information to infer contingencies. Without specific beliefs (e.g., Treatment X is better than Treatment Y), participants hold general ideas about causal candidates (e.g., some treatments are better than others). Here, we have shown that they also hold beliefs about the different roles a variable can play in a three-variate constellation (e.g., a disease may moderate the effect of a treatment, but a patient's last name would not). Therefore, they seem to consider the most likely model to choose a frugal strategy. Sometimes causal assumptions may justify considering an interaction (De Houwer, 2014; Novick & Cheng, 2004; Spellman, 1996), but more often people will look for a simpler model (Lombrozo, 2007, 2016). Thus, when prior beliefs consist of a main effect of variable *x* on variable *y*, rather than an interaction of variable *x* by context, people may switch strategies to infer simple unconditional contingencies. In sum, the present research suggests that contingency inferences from base rates involve top-down processes, and pseudocontingencies – like genuine contingency learning (e.g., Mata et al., 2015; Spellman, 1996) – depend on prior beliefs.

Previous studies of pseudocontingencies have examined diverse contexts, such as treatment-outcome contingencies in hospital wards (Fiedler & Freytag, 2004), social behavior by majorities and minorities in towns (Meiser & Hewstone, 2004), or performance-gender relations in school subjects (Fiedler et al., 2007, Experiment 3). These efforts have highlighted the potential utility of pseudocontingencies for understanding diverse relationships in multiple contexts. However, the role of contextual plausibility in shaping the use of base-rate information for contingency judgments has received less attention. Our findings suggest that contingency judgments can vary significantly depending on whether individuals rely on base rates within specific contexts or on a broader ecological correlation. This variation is particularly evident in situations where one variable varies across contexts while the other remains constant, resulting in a zero ecological correlation but skewed base rates within each context. The current pseudocontingency framework does not provide a clear prediction for contingency judgments in such situations. Future research on pseudocontingencies should therefore consider the content of a context. In real-world scenarios, two base rates will often not vary systematically across contexts, creating situations where the ecological correlation and within-context base rates make different predictions.

Bridging this theoretical exploration to practical application, consider recent research that applied the concept of pseudocontingencies to the perception of the contingency between healthy and tasty foods (Kunz et al., 2023). The authors manipulated the base rates of healthy and tasty foods in two contexts and demonstrated that participants perceive healthy foods to be tastier than unhealthy foods when the base rates of health and taste were skewed in the same (vs. opposite) direction. However, while food healthiness can vary dramatically across contexts, contemporary food environments are predominantly composed of tasty food options (Stein & Keller, 2015). Thus, while the ecological correlation may often suggest no contingency between health and taste at all, people could still infer from the base rates within specific contexts that healthy foods are tastier in one context, but unhealthy foods are tastier in the other. The present research suggests that whether people use the ecological correlation, or the within-context base rates may depend on the plausibility of the context as a moderator of the health-taste contingency. When comparing two different types of restaurants (e.g., a burger restaurant vs. a vegetarian restaurant), it seems plausible that the health-taste contingency depends on the context (the vegetarian restaurant is actually better at preparing healthy, tasty food). However, when comparing different grocery stores (e.g., a supermarket vs. a convenience store), contextual moderation may be less plausible (the supermarket has more healthy food but it is not necessarily tastier than the healthy food at the convenience store).

### Limitations and outlook

This research is not without limitations. To examine contingency inferences in the absence of mechanisms based on true co-occurrence information (i.e., illusory correlations), we used a presentation blocked by the variables *x* and *y*, and base-rate estimates were assessed immediately prior to contingency judgments. This may have made base-rate information particularly salient for contingency judgments, while making genuine contingency approximation very difficult, if not impossible. However, it should be noted that pseudocontingencies were also demonstrated in previous research where joint frequencies were accessible to participants (Eder et al., 2011; Ernst et al., 2019; Fiedler, 2010). Nevertheless, future research may benefit from changes in presentation mode. It is likely that learning conditions (e.g., joint trivariate observations or bivariate observations blocked by context, *x*, or *y*) interact with prior beliefs (main effect of *x* or moderation by context) to determine strategy selection in contingency inference, and thus would be worthy of further investigation.

Another limitation is that our research design cannot fully distinguish between the influence of within-context base rates and ecological correlation on contingency judgments, and future research is needed to better understand these two sources of base rate inference. Our results in Experiment 1 suggest that there is no additive effect of within-context base rates and ecological correlation on contingency judgments when the content of the context makes moderation-by-context plausible. In Experiment 2, we again find no additive effect of both sources of information, but the results suggest that participants' contingency judgments are not driven solely by within-context base rates when moderation was implausible. Thus, participants appear to rely on unconditional contingencies when the content makes a moderation of the context implausible. At this point it is important to note that the size of this interaction effect in Experiment 2 was small and inconclusive given the Bayesian analysis. However, we found a similar pattern of results when analyzing our data in a multi-level model (for details, see OSM).

Notably, the total (or pooled) contingency across contexts was zero in our experiments, and base rates were not skewed at the aggregate level for all participants (cf. Table 1). Thus, the existing difference between consistent and inconsistent base rates when moderation was implausible cannot be attributed to participants completely ignoring within-context base rates in their judgments. Instead, our results are consistent with the idea that people followed the sign of the ecological correlation when moderation-by-context was implausible.

In an exploratory analysis, we provide some indirect evidence that the ecological correlation predicted contingency judgments more strongly when moderation-by-context was implausible. However, because our research design does not allow us to fully disentangle the influence of within-context baseline rates and ecological correlation, the conclusions drawn here remain somewhat speculative. Future research should employ designs in which the ecological correlation is more graded. In the present research, the ecological correlation was always perfectly positive or negative. One possible experiment could expose participants to scenarios in which base rates are consistent across contexts, that is, within-context base rates always imply the same contingency signs. However, and in contrast to our studies, the ecological correlation may always imply the opposite contingency sign (cf., Vogel, Kutzner, et al., 2013). Our research suggests that as long as the context is a plausible moderator of the contingency, participants should not consider the ecological correlation. However, when the context is not a plausible moderator, people should be more likely to use the ecological correlation. Exposing participants to graded ecological correlations could then provide an answer to the question of how much the ecological correlation influences base rate inferences.

## Conclusion

Pseudocontingencies represent a parsimonious strategy to infer the contingency between two variables by associating frequently occurring levels. The parsimony of this strategy is, however, challenged when decision-makers evaluate the same contingency across multiple contexts. Here, we investigated whether people organize base-rate information more efficiently in memory when consistent base rates across contexts allows for the summarization of redundant information or the learning of unconditional contingencies. We found no evidence that base rates were learned more accurately when base rates were consistent versus inconsistent. Instead, replicating previous research (Vogel et al., 2022), we provide evidence that the base rates within contexts are the driving force of pseudocontingencies. However, our research indicates that the integration of base-rate information into judgments is affected by prior beliefs. If justified by prior beliefs, individuals are ready to infer conditional contingencies from base rates *within* each and every context. However, in the absence of specific beliefs, individuals may resort to inferring just one contingency (thus, the same contingency in every context), which follows the sign of the ecological correlation.

## Supplementary Information

Below is the link to the electronic supplementary material.

Supplementary file1 (PDF 611 KB)

## Footnotes

1 In this paper, binary events are categorical, meaning the presence of one level (e.g., policy support) implies the absence of its opposite level (e.g., policy opposition). The categorial nature of the binary events also means that linking policy support for both entities inherently involves linking policy opposition in the same manner.

2 The Simpson Paradox (Simpson, 1951) is a well-known phenomenon in which the pooled contingency across contexts differs from the conditional contingencies within contexts. Research on the Simpson Paradox examines whether people use the pooled unconditional contingency or the conditional contingencies to make judgments.

3 Our expectations were that impoverished learning leads to base-rate estimates that regress towards the mean. For example, a participant who did not learn base rates accurately should give estimates around the scale midpoint.

4 Nineteen participants had no variance in treatment or outcome base rate estimates, so we were unable to calculate an ecological correlation for these participants. Arguably, these participants have an ecological correlation of zero because at least one base rate does not vary across contexts. Therefore, we recoded missing values as zeros. Including or excluding these participants did not affect the results.

## Declarations

### Open practices statement

The experiments and analyses reported here were not preregistered. The data and code for all analyses have been made available on the Open Science Framework and can be accessed at https://osf.io/bxaqy/.
