# Supplementary material for: Contingency inferences from base rates: A parsimonious strategy?
Source: Mem Cognit. 2024 May 6;52(7):1609–25. doi: 10.3758/s13421-024-01567-y (PMC11522045; doi:10.3758/s13421-024-01567-y)
Supplement: Supplementary file 1 — Supplementary file1 (PDF 611 KB) [file 13421_2024_1567_MOESM1_ESM.pdf]

Online Supplementary Materials

Table of Content

- Supporting Information Text
- Table S1 to S6
- Figure S1 to S3
- References

Supporting Information Text

Experiment 1

Due to its nested structure, data from Experiment 1 was additionally analyzed using a multi-level mixed model. Within this model, contingency estimates were subjected to a to a three-way ANOVA with the factors A base rate, B base rate, and consistency. Our hypothesis was that contingency estimates are more positive in contexts were base rates of Politician A's and B's "yes" responses are skewed in the same direction than when they are not. Further, we hypothesized that this pseudocontingency effect will be more pronounced when the base rates are consistent (vs. inconsistent) across contexts. The results of this model are shown in Table S1. All estimated marginal means including confidence intervals are reported in Table S2 and visualized in Figure S1. Additionally, we also run the same model using Bayesian statistics with the *brms* package (Bürkner, 2017). The results of the Bayesian model including credible intervals are reported in Table S3.

As expected, the A base rate × B base rate interaction was significant, $F(1, 159.85) = 26.34$, $p < .001$. Confirming our prediction, contingency estimates were higher when A and B base rates were skewed in the same direction, $M_{\text{high A | high B}} = .13$, $SE = .05$; $M_{\text{low A | low B}} = .05$, $SE = .05$, than when they were not, $M_{\text{high A | low B}} = -.14$, $SE = .05$; $M_{\text{low A | high B}} = -.20$, $SE = .05$. Furthermore, we observed an unexpected effect of the interaction between A base rate and consistency, $F(1, 238) = 4.05$, $p = .045$. Overall, high A (vs. low A) base rates yielded more positive contingency estimates when the base rates were inconsistent ($M_{\text{high A | inconsistent}} = .05$, $SE = .05$; $M_{\text{low A | inconsistent}} = -.09$, $SE = .05$), whereas there was no such difference when the base rates were consistent ($M_{\text{high A | consistent}} = -.07$, $SE = .05$; $M_{\text{low A | consistent}} = -.05$, $SE = .05$). More relevant to our hypothesis, the three-way interaction involving consistency, A base rate, and B base rate, was not significant, $F(1, 159.85) = 0.41$, $p = .522$. Contrary to our prediction, we did not find evidence that the effect of the base rates within contexts is stronger

when the base rates are consistent across contexts. None of the other effects were significant, either, F's < 2.47, p's > .118.

Experiment 2

In Experiment 2, we also conducted an ANOVA on the contingency estimates. That is, we subjected contingency estimates to a four-way ANOVA with the factors treatment base rate, outcome base rate, consistency and moderation plausibility. The results of the ANOVA are shown in Table S4, estimated marginal means are shown in Table S5 and a subsequent Bayesian analysis in reported in Table S6. A visualization of the estimated marginal means is shown in Figure S2.

The analysis revealed two significant effects. Replicating the previous experiment, the treatment base rate × outcome base rate interaction was highly significant, $F(1, 362.97) = 35.53$, $p < .001$. Again, perceived contingencies were more positive if base rates of Treatment X and improved health were skewed in the same direction ($M_{\text{hi treat | hi out}} = .18$, $SE = .04$; $M_{\text{low treat | low out}} = .09$, $SE = .04$) than when they were not ($M_{\text{hi treat | low out}} = -.12$, $SE = .04$; $_{\text{low treat | hi out}} = -.11$, $SE = .04$). Relevant to our research question, the 4-way interaction involving treatment base rate, outcome base rate, the consistency, and the moderation plausibility was significant, too, $F(1, 362.97) = 7.52$, $p = .006$. To better understand this interaction, we broke down effects by conditions of moderation plausibility.

In the high-plausibility condition (Figure S2a), a 2(consistency) × 2(treatment base rate) × 2(outcome base rate) ANOVA yielded results very similar to those from Experiment 1. The treatment base rate × outcome base rate interaction was significant, $F(1, 246.97) = 16.12$, $p < .001$, while the three-way-interaction was not, $F(1, 246.97) = 0.61$, $p = .434$. Thus, independent of the consistency of the base rates across contexts, participants estimated higher contingencies when base rates of Treatment X and improved health were skewed in the same direction ($M_{\text{hi treat | hi out}} = .13$, $SE = .06$; M; $_{\text{low treat | low out}} = .07$, $SE = .06$) than when they were not ($M_{\text{hi treat | low out}} = -.12$, $SE = .05$; $_{\text{low treat | hi out}} = -.11$, $SE = .05$). The only other significant

effect was a main effect of the consistency, $F(1, 95) = 4.10$, $p = .046$, which reflects that contingency estimates were somewhat lower if the base rates were consistent, $M = -.06$, $SE = .04$, than if they were inconsistent, $M = .05$, $SE = .04$. As this effect was independent of whether base rates of Treatment X and improved health were always skewed in the same or opposite direction (which is captured by the three way-interaction), it is irrelevant to our research question. After all, conditional contingency inferences based on the base rates within contexts were sufficient to account for the findings in the condition in which a moderation by context was plausible.

In the low-plausibility condition (Figure S2b), the ANOVA also yielded a significant treatment base rate × outcome base rate interaction, $F(1, 151.10) = 19.95$, $p < .001$. Pertinent to our prediction, the three-way interaction was also significant, $F(1, 151.10) = 8.63$, $p = .004$. Thus, the treatment base rate × outcome base rate interaction depended on the consistency of the base rates across contexts. The other effects in this analysis did not reach significance, *F*`s < 2.77, *p*'s $> .09$. Only when the base rates were consistent did the treatment base rate × outcome base rate interaction reach significance again, $F(1, 48) = 11.31$, $p = .002$. For those cases, base rates of Treatment X and improved health skewed in the same direction evoked more positive contingency judgments ($M_{\text{hi treat | hi out}} = .29$, $SE = .08$; $M;_{\text{low treat | low out}} = .25$, $SE = .08$) than when the base rates were skewed in the opposite direction ($M_{\text{hi treat | low out}} = -.20$, $SE = .10;_{\text{ low treat | hi out}} = -.21$, $SE = .10$). If the base rates were inconsistent, that is, the context moderated the actual contingency, the treatment base rate × outcome base rate interaction was no longer significant, $F(1, 153) = 2.13$, $p = .147$. Hence, estimates for contexts in which base rates of Treatment X and improved health were skewed in the same direction ($M_{\text{hi treat | hi out}} = .17$, $SE = .08$; $M;_{\text{low treat | low out}} = .-.04$, $SE = .08$) did not differ from estimates for contexts in which base rates of Treatment X and improved health were skewed in the opposite direction ($M_{\text{hi treat | low out}} = -.05$, $SE = .08;$ $M_{\text{low treat | hi out}} = -.02$, $SE = .08$).

Table S1

Linear Mixed Model with Contingency Estimates as Dependent Variable in Experiment 1

Variable	df_1, df_2	F	p
A base rate	1, 238	2.46	.118
B base rate	1, 238	0.08	.783
Consistency	1, 78	0.35	.553
**A base rate × B base rate**	**1, 159.85**	**26.34**	**< .001**
**A base rate × Consistency**	**1, 238**	**4.05**	**.045**
B base rate × Consistency	1, 238	0.05	.816
A base rate × B base rate × Consistency	1, 159.85	0.41	.522

Note. df = degrees of freedom. *p*-values less than .05 are considered statistically significant and bolded.

Table S2

Estimated Marginal Means for Linear Mixed Model in Experiment 1

A base rate	B base rate	Consistency	*M*	*SE*	*95% CI*
High	High	Consistent	.06	.07	[-.08, .20]
Low	High	Consistent	-.15	.07	[-.30, -.05]
High	Low	Consistent	-.19	.07	[-.34, -.04]
Low	Low	Consistent	.06	.07	[-.08, .19]
High	High	Inconsistent	.20	.07	[.07, .33]
Low	High	Inconsistent	-.24	.07	[-.37, -.11]
High	Low	Inconsistent	-.10	.07	[-.23, .03]
Low	Low	Inconsistent	.05	.07	[-.08, .18]

Note. M = mean; *SE* = standard error; *CI* = confidence interval.

Table S3

Bayesian Multilevel Analysis of Contingency Estimates in Experiment 1

Variable	Estimate	*SE*	95% CI
Intercept	-0.04	0.03	[-0.10, 0.02]
A base rate	-0.03	0.02	[-0.07, 0.01]
B base rate	-0.01	0.02	[-0.05, 0.04]
Consistency	-0.02	0.03	[-0.08, 0.04]
**A base rate × B base rate**	**0.13**	**0.03**	[0.08, 0.18]
A base rate × Consistency	0.04	0.02	[-0.00, 0.08]
B base rate × Consistency	-0.01	0.02	[-0.04, 0.03]
A base rate × B base rate × Consistency	-0.02	0.03	[-0.07, 0.03]

Note. *CI* = credible interval; Rhat values were at the convergence criterion of 1.00 for all parameters, indicating successful convergence of the model. For the categorical variables “Treat base rate”, “Out base rate” and “Consistency” sum-to-zero contrasts were applied with “Low”, “Low” and “Consistent” coded as first level (-1) and “High”, “High”, “Inconsistent” and “Implausible” coded as second level (1). Estimates with credible intervals that do not contain zero are bolded.

Table S4

Linear Mixed Model with Contingency Estimates as Dependent Variable in Experiment 2

Variable	df_1, df_2	F	p
Treat base rate	1, 590	1.50	.221
Out base rate	1, 590	2.84	.093
Consistency	1, 194	0.97	.326
Plausibility	1, 194	0.44	.508
**Treat base rate × Out base rate**	**1, 362.97**	**35.53**	**< .001**
Treat base rate × Consistency	1, 590	1.66	.198
Out base rate × Consistency	1, 590	0.01	.941
Treat base rate × Plausibility	1, 590	.209	.648
Out base rate × Plausibility	1, 590	.171	.679
Consistency × Plausibility	1, 194	1.68	.196
Treat base rate × Out base rate × Consistency	1, 362.97	3.06	.081
Treat base rate × Out base rate × Plausibility	1, 362.97	0.75	.386
Treat base rate × Consistency × Plausibility	1, 590	.053	.818
Out base rate × Consistency × Plausibility	1, 590	2.27	.132
**Treat base rate × Out base rate × Consistency × Plausibility**	**1, 362.97**	**7.52**	**.006**

Note. df = degrees of freedom. *p*-values less than .05 are considered statistically significant and bolded.

Table S5

Estimated Marginal Means for Linear Mixed Model in Experiment 2

Treat base rate	Out base rate	Consistency	Plausibility	*M*	*SE*	*95% CI*
High	High	Consistent	Plausible	.06	.10	[-.13, .24]
Low	High	Consistent	Plausible	-.09	.08	[-.25, .06]
High	Low	Consistent	Plausible	-.21	.08	[-.36, -.05]
Low	Low	Consistent	Plausible	-.01	.10	[-.20, .18]
High	High	Inconsistent	Plausible	.21	.07	[.07, .36]
Low	High	Inconsistent	Plausible	-.12	.07	[-.26, .03]
High	Low	Inconsistent	Plausible	-.04	.07	[-18, .11]
Low	Low	Inconsistent	Plausible	.14	.07	[-.00, .29]
High	High	Consistent	Implausible	.29	.08	[.14, .44]
Low	High	Consistent	Implausible	-.21	.09	[-.38, -.03]
High	Low	Consistent	Implausible	-.20	.09	[-.38, -.03]
Low	Low	Consistent	Implausible	.25	.08	[10, .41]
High	High	Inconsistent	Implausible	.17	.07	[.03, .32]
Low	High	Inconsistent	Implausible	-.02	.07	[-.16, .13]
High	Low	Inconsistent	Implausible	-.05	.07	[-.29, .10]
Low	Low	Inconsistent	Implausible	-.04	.07	[-.18, .11]

Note. M = mean; *SE* = standard error; *CI* = confidence interval.

Table S6

Bayesian Multilevel Analysis of Contingency Estimates in Experiment 2

Variable	Estimate	*SE*	95% CI
Intercept	0.01	0.02	[-0.04, 0.06]
Treat base rate	-0.02	0.02	[-0.05, 0.01]
Out base rate	-0.03	0.02	[-0.06, 0.00]
Consistency	-0.02	0.02	[-0.08, 0.03]
Plausibility	-0.02	0.03	[-0.07, 0.03]
**Treat base rate × Out base rate**	**0.13**	**0.02**	**[0.09, 0.17]**
Treat base rate × Consistency	0.02	0.02	[-0.01, 0.05]
Out base rate × Consistency	0.00	0.02	[-0.03, 0.03]
Treat base rate × Plausibility	0.01	0.02	[-0.02, 0.04]
Out base rate × Plausibility	0.01	0.02	[-0.03, 0.04]
Consistency × Plausibility	-0.03	0.02	[-0.08, 0.02]
Treat base rate × Out base rate × Consistency	0.04	0.02	[-0.00, 0.08]
Treat base rate × Out base rate × Plausibility	-0.02	0.02	[-0.06, 0.02]
Treat base rate × Consistency × Plausibility	0.00	0.02	[-0.03, 0.04]
Out base rate × Consistency × Plausibility	-0.03	0.02	[-0.06, 0.01]
**Treat base rate × Out base rate × Consistency × Plausibility**	**-0.06**	**0.02**	**[-0.10, -0.02]**

Note. *CI* = credible interval; Rhat values were at the convergence criterion of 1.00 (or 1.01) for all parameters, indicating successful convergence of the model. For the categorical variables “Treat base rate”, “Out base rate”, “Consistency” and “Plausibility” sum-to-zero contrasts were applied with “Low”, “Low”, “Consistent” and “Plausible” coded as first level (-1) and “High”, “High”, “Inconsistent” and “Implausible” coded as second level (1). Estimates with credible intervals that do not contain zero are bolded.

Figure S1

Results from Experiment 1

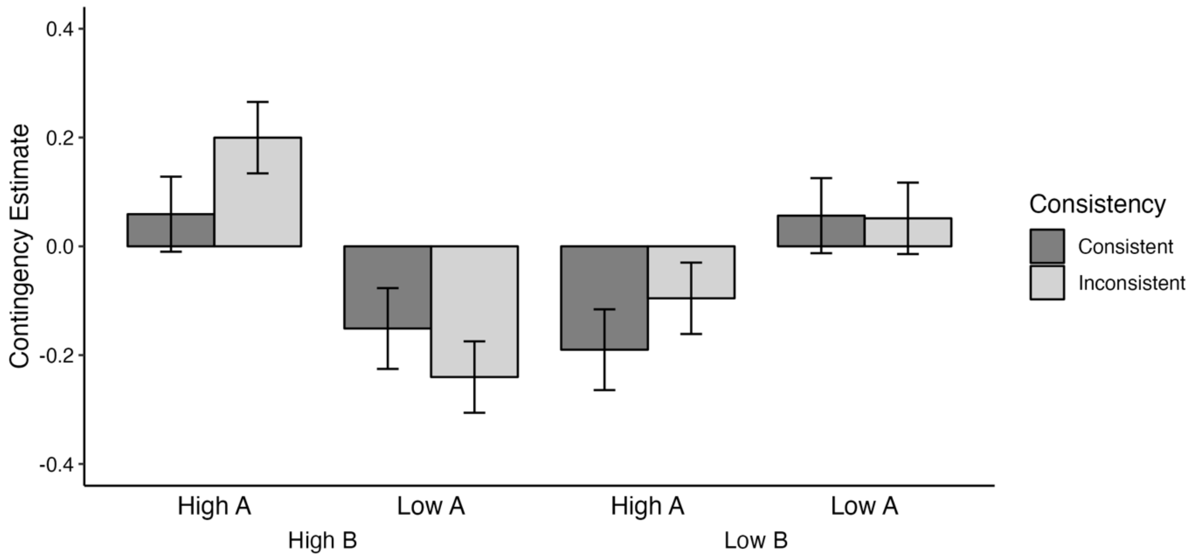

Note. Estimates for contingency between A (yes responses by Politician A) and B (yes responses by Politician B) as function of A and B base rates. The consistency of the base rates across contexts varied between participants. Error bars represent standard errors.

Figure S2

Results from Experiment 2

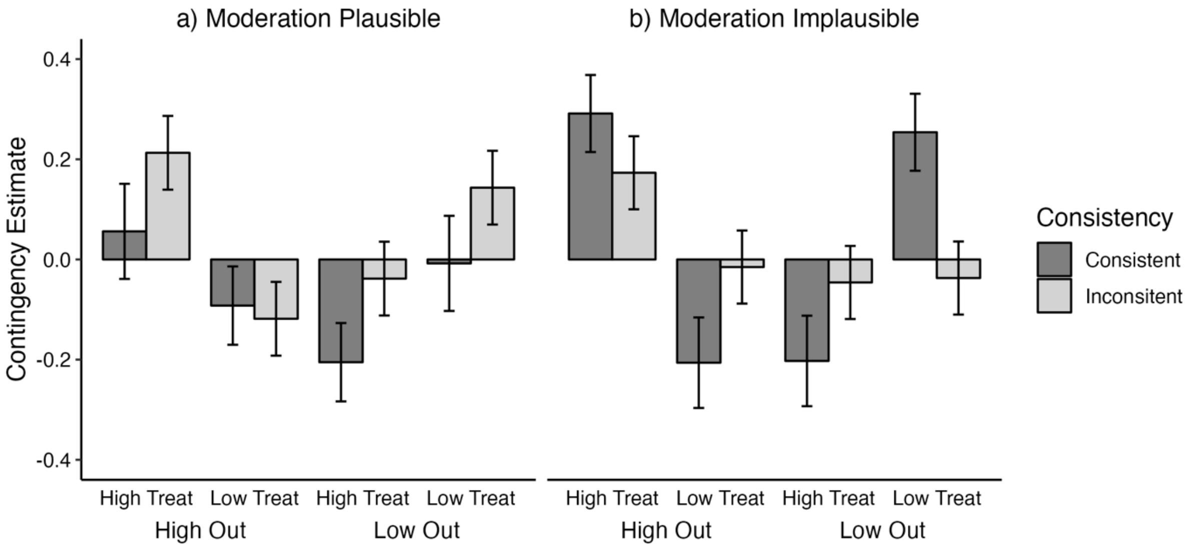

Note. Estimates for treatment-outcome contingency as a function of treatment and outcome base rates. The consistency and the plausibility of the context as a moderator of treatment-outcome contingencies varied between participants. Error bars represent standard errors.

Figure S3

Effect of Perceived Ecological Correlation and Context Moderation (Plausible vs. Implausible) on Inferred Domain-Wise Contingency Estimates in Experiment 2

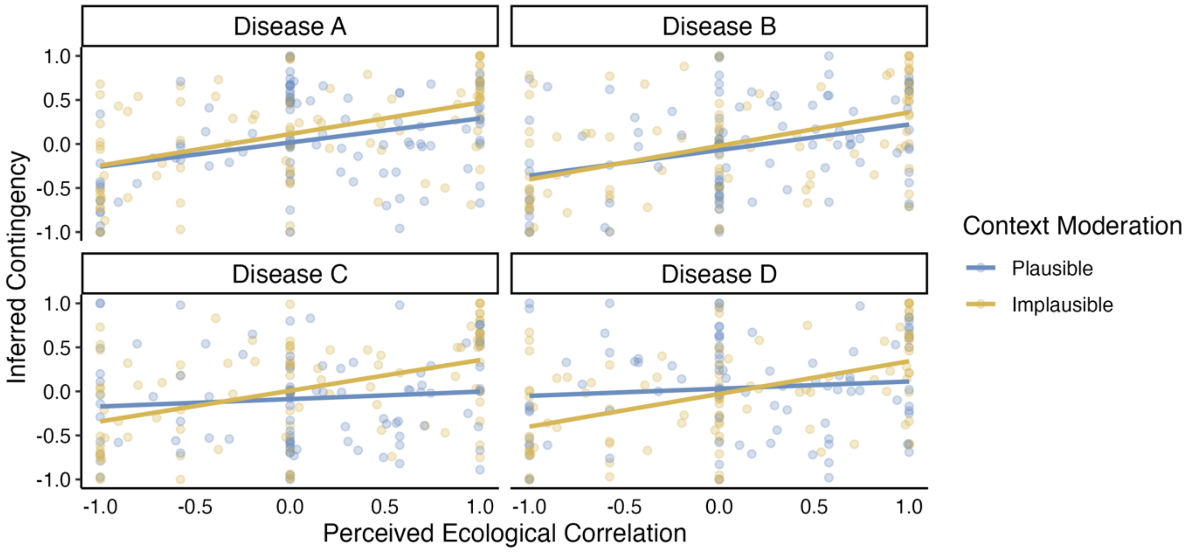

References

Bürkner, P. (2017). Advanced Bayesian multilevel modeling with the R package brms. *Journal of Statistical Software*, *80*(1), 1–28. https://doi.org/10.18637/jss.v080.i01
